# Epidemiological analysis of death among patients on maintenance hemodialysis: results from the beijing blood purification quality Control and Improvement Center

**DOI:** 10.1186/s12882-023-03271-6

**Published:** 2023-08-15

**Authors:** Jing Liu, Huixian Zhang, Zongli Diao, Wang Guo, Hongdong Huang, Li Zuo, Wenhu Liu

**Affiliations:** 1 Department of Nephrology, Beijing Friendship Hospital, Capital Medical University, Beijing, China; 2 Department of Nephrology, Peking University People’s Hospital, Peking University, Beijing, China

**Keywords:** Epidemiology, Cause of death, Maintenance hemodialysis

## Abstract

**Background:**

China has the largest number of patients on maintenance hemodialysis (MHD) worldwide. Despite continuous improvements in hemodialysis techniques, patients on MHD have a higher mortality rate than the general population. Understanding the characteristics of death in this population can better promote clinical practice, thereby improving patients’ survival.

**Methods:**

We collected demographic and clinical data for patients on MHD registered in the Beijing Blood Purification Quality Control and Improvement Center database from 2014 to 2020. The annual mortality rate was calculatedand the primary cause of end-stage renal disease (ESRD), dialysis vintage, and cause of death among deceased patients were analyzed.

**Results:**

(1) 24,363 patients on MHD were included, of which 6,065 patients died from 2014 to 2020. The annual mortality rate fluctuated between 7.4% and 8.0%. The median age of death was 70.0 (60.8–79.0) years and the male to female ratio was 1.27:1 (2). The top three primary causes of ESRD in deceased patients were chronic glomerulonephritis (CGN), diabetic nephropathy (DN), and hypertensive nephropathy (HN). Comparison of the annual mortality rate showed DN > HN > CGN (3). The median dialysis vintage of deceased patients was 3.7 (1.8–6.9) years, which slowly increased annually. Patients with diabetes had a shorter dialysis vintage than patients without diabetes (3.4 vs. 4.1 years, Z = 8.3, P < 0.001) (4). The major causes of death were cardiovascular disease (20.2%), sudden death (18.1%), infection (17.9%), and cerebrovascular disease (12.6%). Proportions of death from cardiovascular disease, infection, and sudden death were higher in patients with diabetes (22.2%, 20.2%, and 20.0%) than patients without diabetes (18.4%, 15.8%, and 16.3%). Sudden death was the leading cause of death in young (18–44 years; 27.0%) and middle aged (45–64 years; 20.8%) patients, whereas infection was the leading cause of death in patients aged ≥ 75 years (24.5%).

**Conclusion:**

The annual mortality rate of patients on MHD in Beijing was relatively stable from 2014 to 2020. Sudden death was more likely to occur in young and middle-aged patients, and more patients aged ≥ 75 years died from infections.

## Background

Kidney disease is an important disease affecting human health globally. Currently, the prevention and control of kidney disease in China still faces serious challenges. The incidence of kidney disease in China is high, with approximately 120 million patients with chronic kidney disease (CKD), and 1 to 2 million new cases of uremia each year [1]. The number of Chinese patients with CKD and ESRD are increasing annually, bringing a heavy burden on China’s medical and health resources. Therefore, kidney disease has become a major disease and an important public health issue that affects the national health of China. Today, hemodialysis remains the main blood purification treatment for ESRD nationwide. The China Kidney Disease Network 2016 Annual Data Report showed that patients on hemodialysis accounted for 91.94% of all patients on dialysis [2]. The number of patients on maintenance hemodialysis (MHD) reached 735,000 in 2021 in China, ranking the highest worldwide [1]. Continuous technical improvement of blood purification has improved the quality of patients’ life and prolonged their survival time. However, compared with the general population, patients on hemodialysis have a 70% shorter life expectancy and a high mortality rate [3]. Therefore, investigating epidemiological characteristics of deceased patients and factors influencing death and taking appropriate measures to improve daily clinical practice, which will minimize the risk for death, will be beneficial for their survival and prognosis.

The present study retrospectively analyzed the death status of patients on MHD and aimed to clarify the characteristics of death among this population during the last 7 years in Beijing, to provide useful information for clinical practice.

## Methods

Data for this study were obtained from the Beijing Blood Purification Quality Control and Improvement Center (BJBPQCIC), which manages all hemodialysis centers except the 10 military dialysis centers in Beijing. Since 2007, the BJBPQCIC has used an electronic data collection system to collect patient-level data. To improve data integrity, the platform generates an integrity degree (%) for each data variable, which is fed back to each center during the annual quality control inspection. The centers are urged to improve the integrity of data in the next year. Data integrity has therefore continuously improved, and the degree of data integrity for the variables involved in this paper was above 90% in 2020.

### Study Population

All patients on MHD who were registered in the BJBPQCIC database from 1st January 2014 to 31st December 2020 were included in our analysis. The inclusion criteria were patients aged ≥ 18 years of either sex with a dialysis vintage > 90 days. Exclusion criteria were lack of important data, including date of birth, date of first dialysis, outcome (death, peritoneal dialysis, kidney transplantation, transfer, renal function recovery, withdrawal), or date of outcome.

### Study methods

This was a cross-sectional study. Demographic and clinical data were collected from the BJBPQCIC database, including date of birth, sex, primary cause of ESRD, date of first dialysis, outcome and date of outcome, cause of death, and comorbidities. Data collection, collation, and analysis were performed by dedicated personnel.

The annual mortality rate was calculated as follows. The annual number of deceased patients on MHD / the annual number of patients on MHD (person-years).

The dialysis vintage of deceased patients (years) refers to the interval between the date of first dialysis and the date of death.

Normally distributed data were expressed as mean ± standard deviation, and non-normally distributed data were expressed as median (quartile) (M [P25%–P75%]). Categorical variables were expressed as frequency (percentage).The dialysis vintages between the two groups (with or without diabetes) were compared by rank sum test. All analyses were conducted using SPSS 26.0 software (IBM Corp., USA).

## Results

**Fig. 1:**
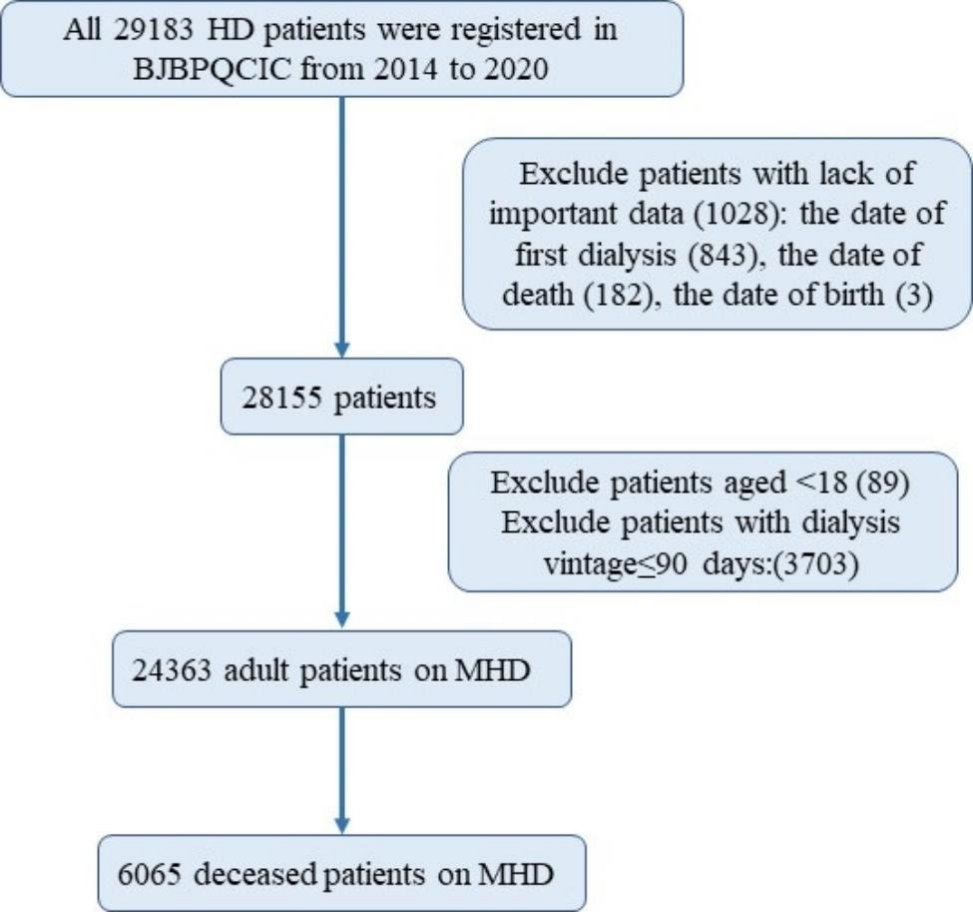
Process of screening patients for this study

In total, 29,183 patients were registered in the BJBPQCIC database between 2014 and 2020. Based on our inclusion and exclusion criteria, we eventually included 24,363 patients undergoing MHD (Fig. 1).

### Demographic data and the Annual Mortality Rate

There were 24,363 patients on MHD, with a male-to-female ratio of 1.41:1. Of these, 6,065 patients died, and the annual mortality rate fluctuated between 7.4% and 8.0%. Among the deceased patients, the median age of death was 70.0 (60.8–79.0) years, patients aged ≥ 65 years accounted for 63.9%, and the male-to-female ratio was 1.27:1, which remained relatively stable each year (Table 1).

**Table 1:** Annual mortality rate and demographic data for MHD patients who died in Beijing from 2014 to 2020

year	number of MHD patients (person-years)	number of deaths	mortality rate (%)	age of death (years)	sex of death n (%)		transfer to PD	transfer to KT
					male	female		
2014	9640	716	7.4	71.6(60.4–78.8)	373(52.1)	343(47.9)	66 (0.7%)	432 (4.5%)
2015	10,289	792	7.7	70.6(60.0-79.2)	416(52.5)	376(47.5)	64(0.6%)	451 (4.4%)
2016	10,793	849	7.9	69.6(60.4–79.2)	486(57.2)	363(42.8)	46(0.4%)	436 (4.0%)
2017	11,350	896	7.9	69.8(60.3–78.9)	481(53.7)	415(46.3)	46 (0.4%)	406 (3.6%)
2018	11,743	934	8.0	70.3(61.2–79.5)	532(57.0)	402(43.0)	33 (0.3%)	344 (2.9%)
2019	12,173	920	7.6	70.5(61.0-79.6)	542(58.9)	378(41.1)	25 (0.2%)	223 (1.8%)
2020	12,450	958	7.7	69.0(61.8–78.3)	569(59.4)	389(40.6)	15 (0.1%)	120 (1.0%)

Data are n (%) or median (P25%–P75%). MHD: maintenance hemodialysis; PD: peritoneal dialysis; KT: kidney transplantation

### Primary cause of ESRD

**Fig. 2:**
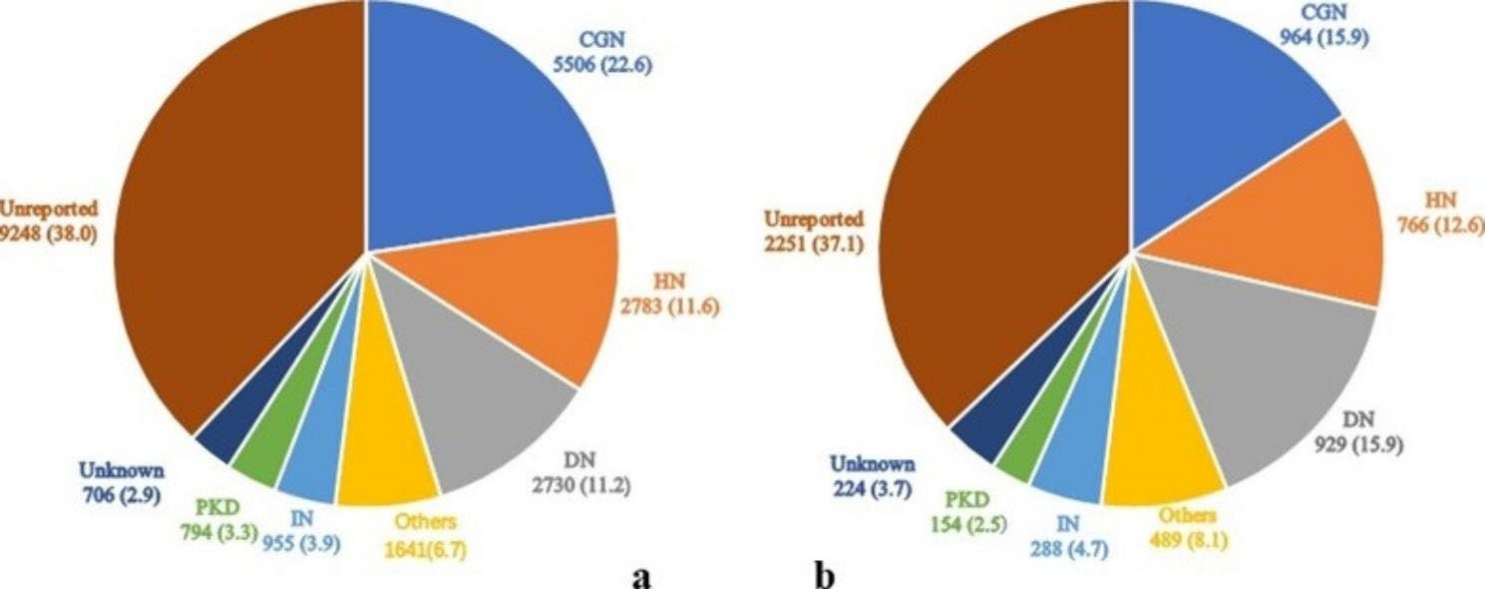
Primary cause of end-stage renal disease among patients on maintenance hemodialysis in Beijing from 2014 to 2020. Data are n (%). a: all patients (N = 24,363), b: deceased patients (N = 6,065). CGN: chronic glomerulonephritis; DN: diabetic nephropathy; HN: hypertensive nephropathy; IN: interstitial nephropathy; PKD: polycystic kidney disease

Among the 24,363 patients, the top three primary causes of ESRD were chronic glomerulonephritis (CGN) (n = 5,506, 22.6%), hypertensive nephropathy (HN) (n = 2,783, 11.6%), and diabetic nephropathy (DN) (n = 2,730, 11.2%). Among the 6,065 deceased patients, the top three primary causes of ESRD were CGN (n = 964, 15.9%), DN (n = 929, 15.3%), and HN (n = 766, 12.6%) (Fig. 2).

**Fig. 3:**
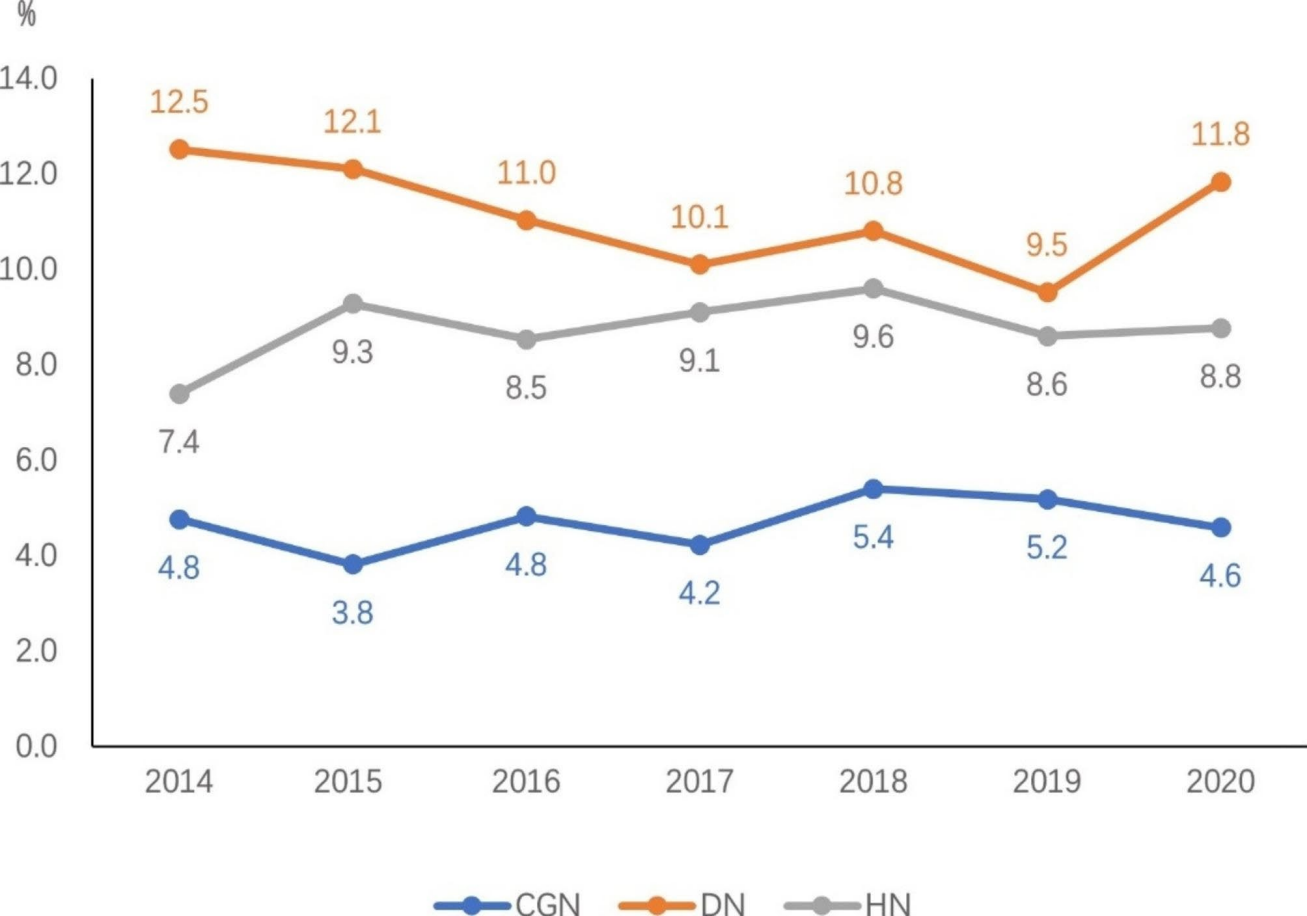
Annual mortality rate of patients on maintenance hemodialysis in Beijing from 2014 to 2020 by the top three primary causes of end-stage renal disease (%). CGN: chronic glomerulonephritis; DN: diabetic nephropathy; HN: hypertensive nephropathy

Comparison of the annual mortality rate according to the top three primary causes of ESRD showed that DN > HN > CGN (Fig. 3).

### Dialysis Vintage

The median dialysis vintage of deceased patients was 3.7 (1.8–6.9) years; 38.2% had a dialysis vintage ≥ 5 years, and 12.3% had a dialysis vintage ≥ 10 years. The proportions of patients with dialysis vintages of < 1 year and 1–3 years decreased annually, whereas those with dialysis vintages of 5–10 years and more than 10 years increased annually. The annual median dialysis vintage showed a slow increase over time (Table 2).

**Table 2:** Number of patients grouped by dialysis vintages among patients on MHD who died in Beijing who died from 2014 to 2020

year	the number of patients in each dialysis vintage group (n, %)					the median dialysis vintage(years)
	0.25–0.99	1.0–2.9	3.0–4.9	5.0–9.9	≥ 10.0	
2014	100 (14.0)	226 (31.6)	140 (19.6)	174 (24.3)	76 (10.6)	3.4 (1.7–6.6)
2015	112 (14.1)	251 (31.7)	147 (18.6)	215 (27.1)	67 (8.5)	3.3 (1.6–6.5)
2016	111 (13.1)	238 (28.0)	177 (20.8)	221(26.0)	102 (12.0)	3.8 (1.8–6.8)
2017	122 (13.6)	242 (27.0)	175 (19.5)	244 (27.2)	113 (12.6)	4.0 (1.7–7.0)
2018	114 (12.2)	272 (29.1)	202 (21.6)	227 (24.3)	119 (12.7)	3.8 (1.8–6.8)
2019	119 (12.9)	252 (27.4)	182 (19.8)	233 (25.3)	134 (14.6)	3.9 (1.8–7.1)
2020	113 (11.8)	268 (28.0)	190 (19.8)	254 (26.5)	133(13.9)	4.0 (1.8–7.3)
Total	791 (13.0)	1749 (28.8)	1213 (20.0)	1568 (25.9)	744(12.3)	3.7 (1.8–6.9)

Data are n (%) or median (P25%–P75%). MHD: maintenance hemodialysis

**Fig. 4:**
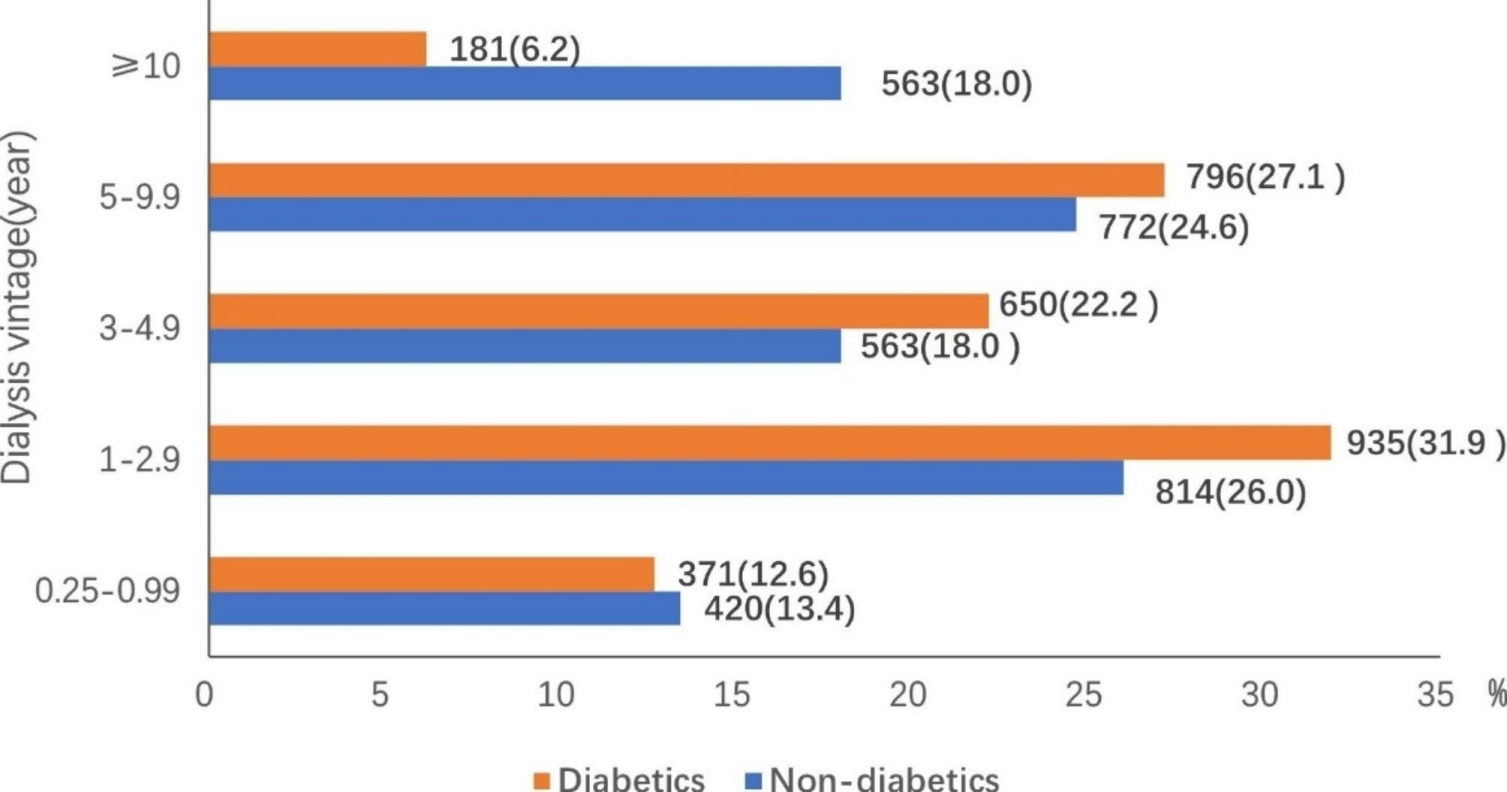
Dialysis vintage of patients on maintenance hemodialysis with or without diabetes who died in Beijing from 2014 to 2020. Data are n (%) (N = 6,065: patients with diabetes n = 2,933, patients without diabetes n = 3,132)

The median dialysis vintage was 3.4 (1.7– 5.9) years in patients with diabetes and 4.1 (1.8–8.3) years in patients without diabetes (Z = 8.3, P < 0.001). After further grouping by dialysis vintage, we found that the proportion of patients with a dialysis vintage ≥ 10 years was significantly lower among patients with diabetes (6.2%) than patients without diabetes (18.0%) (Fig. 4).

### Cause of death

**Fig. 5:**
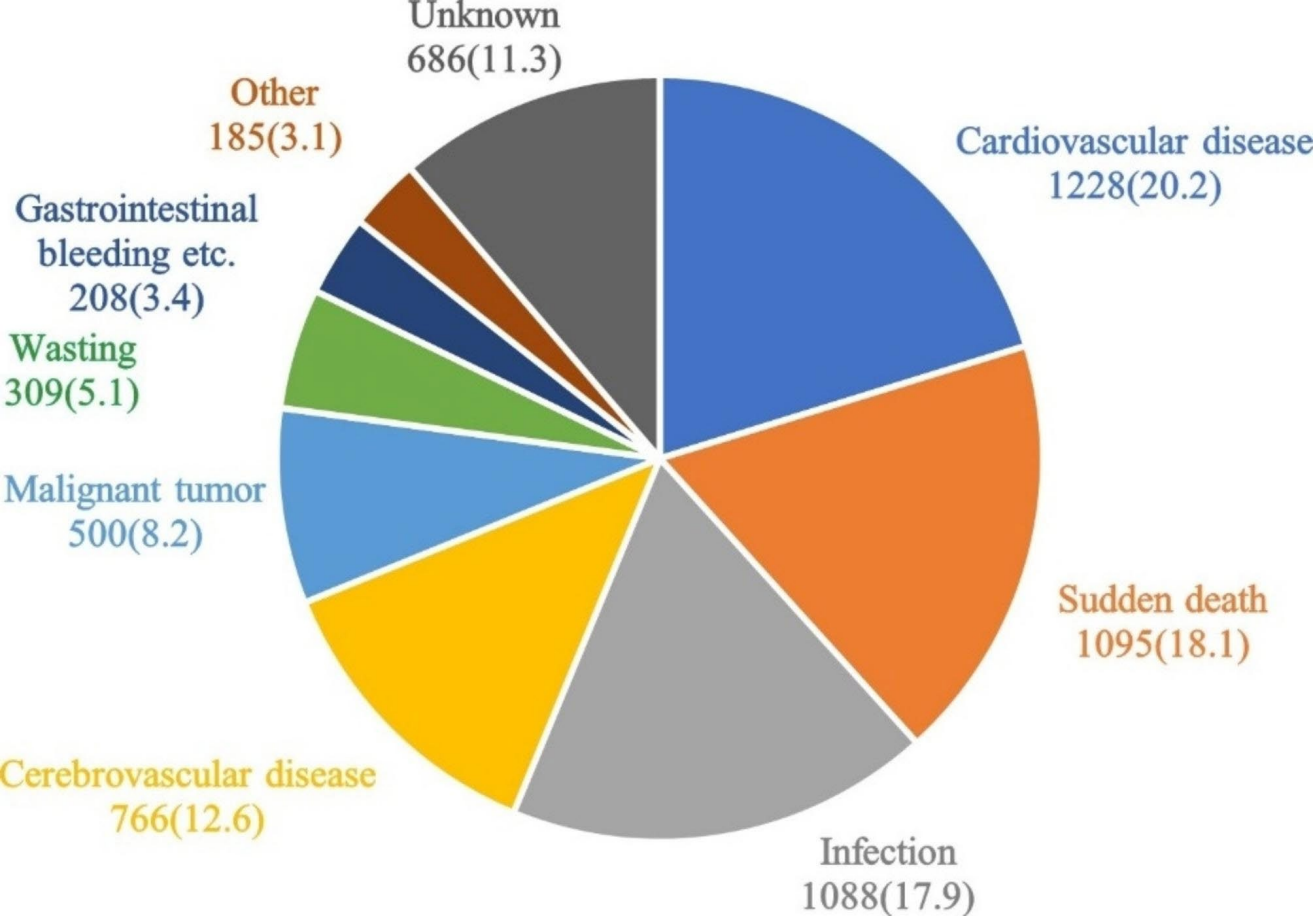
Causes of death among patients on maintenance hemodialysis in Beijing who died from 2014 to 2020. Data are n (%) (N = 6,065)

The leading cause of death was cardiovascular disease (n = 1,228, 20.2%), followed by sudden death (n = 1,095, 18.1%) and infection (n = 1,088, 17.9%) (Fig. 5).

**Fig. 6:**
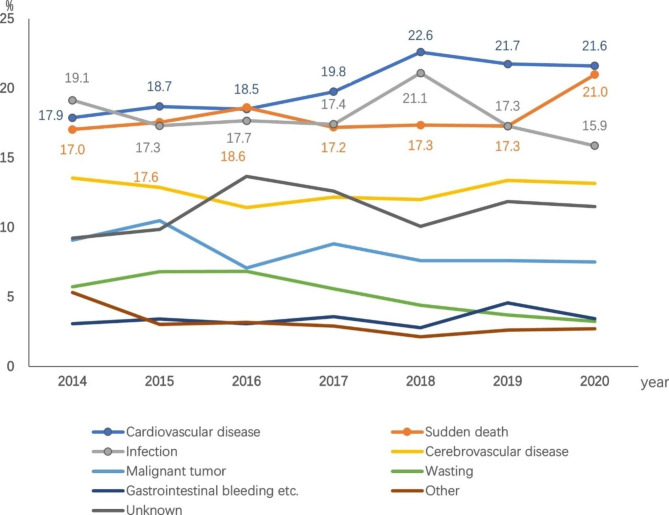
Proportion of causes of death in each year among patients on maintenance hemodialysis in Beijing who died from 2014 to 2020 (%)

The proportions of each cause of death annually from 2014 to 2020 are shown in Fig. 6. The top cause of death each year fluctuated among cardiovascular disease, sudden death, and infection.

**Fig. 7:**
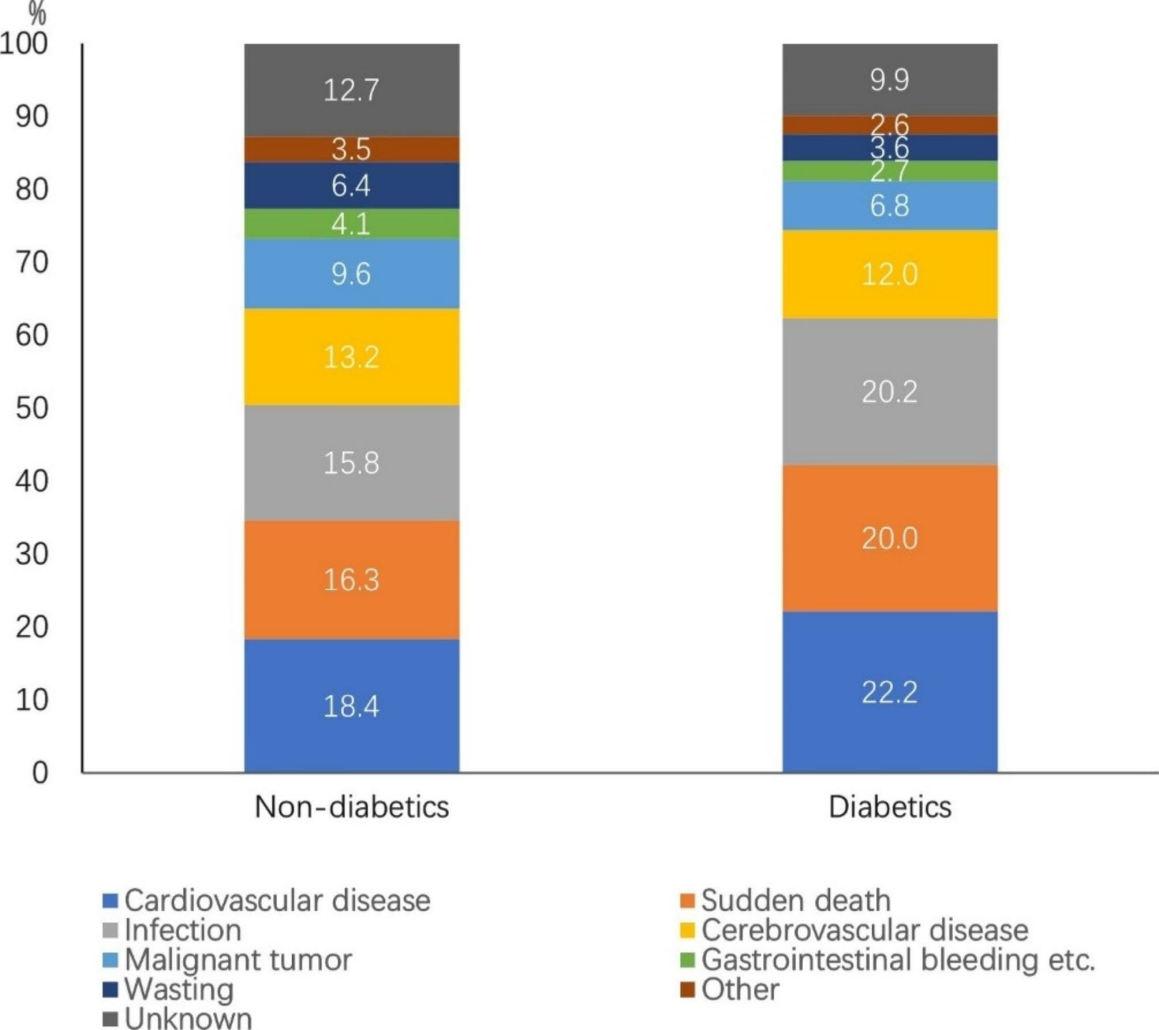
Causes of death among patients with or without diabetes on maintenance hemodialysis in Beijing who died from 2014 to 2020 (%) (N = 6,065: patients without diabetes n = 3,132, patients with diabetes n = 2,933)

The proportions of death from cardiovascular disease, sudden death, and infection were higher among patients with diabetes than patients without diabetes (Fig. 7).

**Fig. 8:**
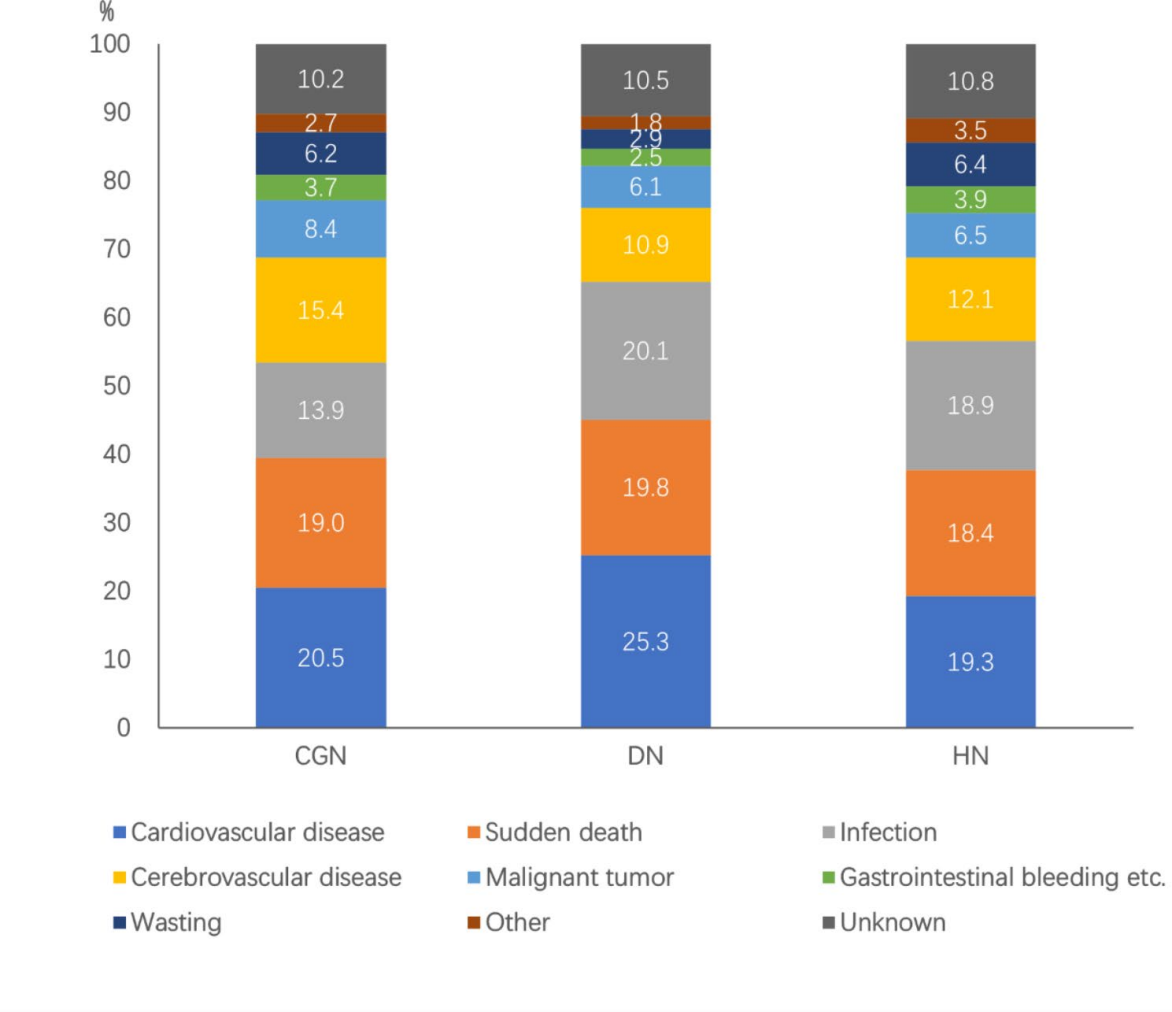
Causes of death among patients on maintenance hemodialysis in Beijing who died from 2014 to 2020 (%) grouped by primary cause of end-stage renal disease (CGN n = 964, DN n = 929, HN n = 766). CGN: chronic glomerulonephritis; DN: diabetic nephropathy; HN: hypertensive nephropathy

The leading cause of death in the three groups of patients with CGN, DN, or HN as the primary cause of ESRD was cardiovascular disease. The proportions of death from cardiovascular disease, sudden death, and infection were higher among patients with DN than patients with CGN and HN (Fig. 8).

**Fig. 9:**
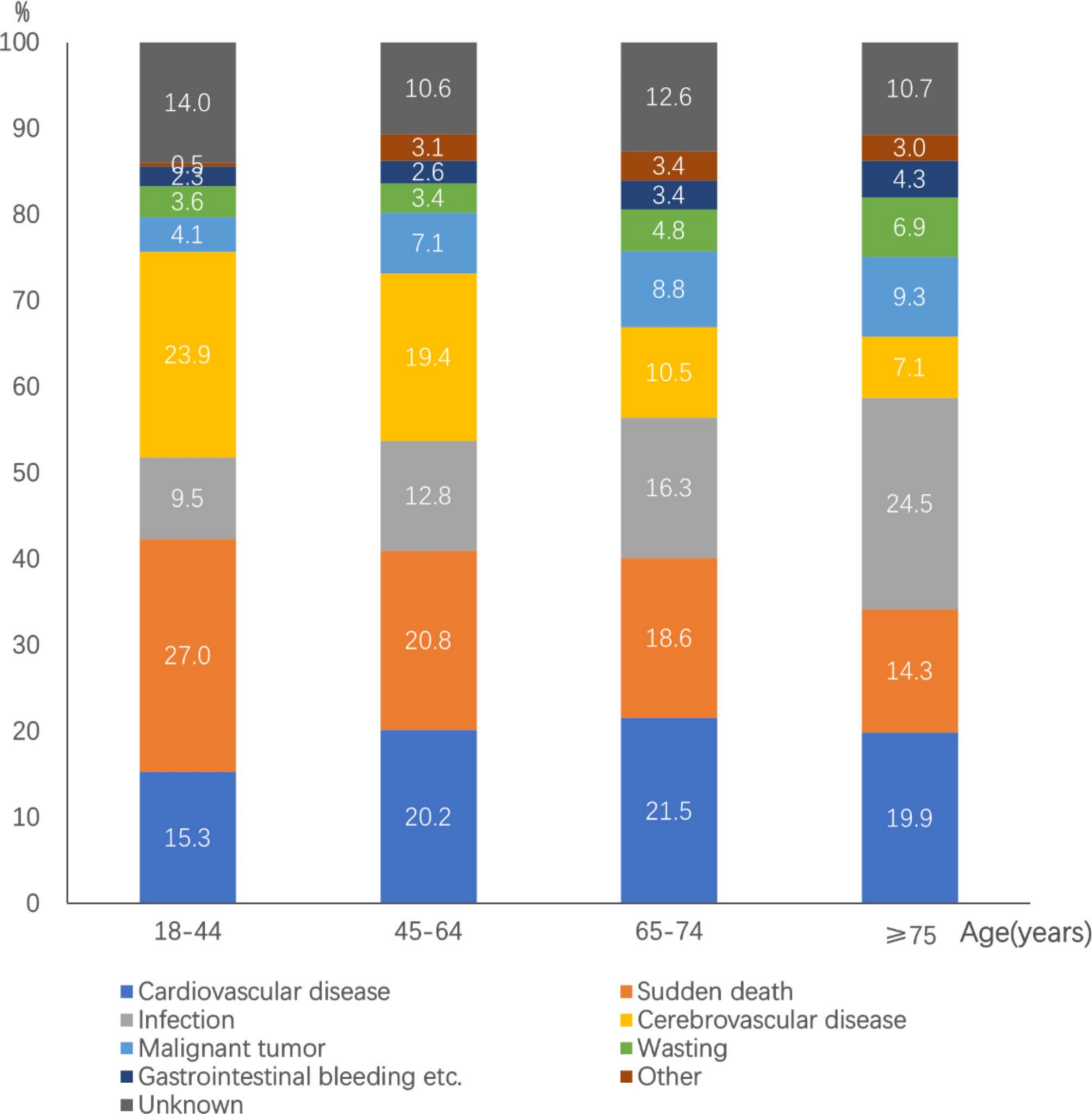
Causes of death among patients on maintenance hemodialysis in Beijing who died from 2014 to 2020 (%) grouped by age (18–44 years, n = 222; 45–64 years, n = 1,964; 65–74 years, n = 1,634; ≥75 years, n = 2,245)

The top three causes of death differed among different age groups, and the proportions of death from sudden death and cerebrovascular disease were higher in young (18–44 years) and middle-aged (45–64 years) patients than older patients (≥ 65 years). Sudden death was the leading cause of death in young and middle-aged patients. The proportions of death from cardiovascular disease and infection were higher in older patients (≥ 65 years). Cardiovascular disease was the leading cause of death in the group aged 65–74 years, and infection was the leading cause of death in patients aged ≥ 75 years (Fig. 9).

## Discussion

This study retrospectively analyzed the death status of patients on MHD in Beijing from 2014 to 2020. We found that the annual mortality rate was relatively stable. The dialysis vintage of deceased patients increased annually, and the main causes of death were cardiovascular disease, sudden death, infection, and cerebrovascular disease. The proportions of death from sudden death and infection in all deceased patients increased significantly compared with those in previous periods (2007–2011). Sudden death was the leading cause of death in young and middle-aged patients, while more patients aged ≥ 75 years died from infections. Diabetes, whether as a comorbidity or the primary cause of ESRD, showed a high mortality and a short survival time.

This study showed that the annual mortality rate of patients on MHD in Beijing from 2014 to 2020 ranged from 7.4% to 8.0% and remained basically stable compared with the previously reported rate for 2007–2010 of 7.4–9.0% [4]. This was close to previously reported rates for Wuhan (10.3–7.1%) [5] and Shanghai (4.6–8.4%) [6] in China and in Japan (9.6–10.0%, 2014–2018) [7]. It was much lower than the rate reported for the United States (16.7–15.9%,2014–2019) [8]. Even compared with the mortality rate of Asian Americans (13.2–13.1%) [8], the rate in our study still showed a significant survival advantage. This result was consistent with a previous study focused on the difference in mortality rate between patients on MHD in Beijing and the United States, which suggested that the survival advantage of Beijing patients may be related to race and the clinical practice pattern [4]. Kawaguchi et al. investigated the relationship between the frequency and duration of patient–doctor contact (PDC) and clinical outcomes during hemodialysis treatment [9]. The result showed that more frequent and longer PDC was inversely associated with all-cause mortality and the rate of first hospitalization, and the role of physicians in the team of healthcare professionals was critical for improving the quality of hemodialysis [9]. In Beijing, doctors in the dialysis center are relatively permanent and have frequent contact with patients. According to our experience, the PDC frequency is at least 8–12 times/month, which is much higher than the 4 times/month reported by Kawaguchi et al [9]. Whether formulating and adjusting dialysis prescriptions before and after dialysis, or making ward rounds to see patients during dialysis, doctors in Beijing remain actively involved. This clinical practice pattern is more refined and individualized, which is conducive to timely detection and treatment of situations that may endanger patients’ lives. In addition, there is a broad consensus that differences in hemodialysis vascular access use are a significant part of mortality differences across countries [10]. Our study did not analyze the current situation of vascular access, but the results of the Dialysis Outcomes and Practice Patterns Study Phase 5(DOPPS5) conducted in China (2012–2015) showed the utilization rate of arterio-venous fistula in patients on MHD in Beijing was 87.2% [11], which was lower than the proportion in Japan (95%) [12] but higher than that in the United States (63.9–65.6%) [8]. Therefore, this could also be a reason for the above mentioned difference in mortality rates.

It should be noted that, one cause of death in the United States Renal data System was withdrawal, which accounted for approximately 15% of the total deaths [8]. There was no related death record in our database for deceased patients who had withdrawn from dialysis, therefore, our study might have underestimated the overall mortality rate. However, we believe that the number of patients who withdrew from hemodailysis was small because ESRD has been covered by medical insurance in Beijing since 2004, and the government provides economic security for patients. Therefore, few patients on MHD in Beijing quit treatment for economic reasons. In this study, 101 patients withdrew from hemodialysis for economic reasons, but we did not know whether they died. Even if they were all assumed to have died and were included in the deceased patient group, it would only account for 1.6% of the total deaths. In addition, few patients quit dialysis because of serious illness. Therefore, this confounding factor only had a slight effect on the actual mortality rate in our study.

The differences in mortality rates among patients on MHD in different countries or regions are also influenced by factors such as age, primary cause of ESRD, and comorbidities. For the period from 2014 to 2018, the patients on hemodialysis in Beijing and the United States were younger (33.6 − 40.4% and 45.5 − 47.0% [8]  ≥ 65 years old ,respectively) than those in Japan(63.8 − 67.9%≥ 65 years old [7]; regarding the primary cause of ESRD, the proportion of patients on dialysis with DN was substantially lower in Beijing (14.8–17.6%) than in the United States (45.5–47.0%) [8] and Japan (38.1–39.0%) [7]. Although comorbidities are also an important factor affecting the survival time , we were unable to compare the comorbidities of our patients on MHD with those in Japan and the United States because of the incomplete information in our database. These differences in population characteristics will affect the comparison of mortality rates between regions.

Cardiovascular disease has long been the leading cause of death in patients on dialysis, and this study showed the same results (20.2%). This proportion was decreased compared with previous data for Beijing (2007–2011), from 25.4 to 20.2% [13]. However, the proportions of death from infection and sudden death both increased, with infection increasing from 13.1 to 17.9% and sudden death significantly increasing from 7.4 to 18.1%. The proportion of sudden death in our study is similar to that reported in Sichuan Province [14] and Shanxi Province [15] in China, but is higher than that in Japan [7] and lower than that in the United States [8]. In our study, 1,095 cases of sudden death were reported, including 858 cases of sudden cardiac death (78.4%). Sudden death was the leading cause of death in young(18–44 years) and middle-aged patients (45–64 years), and its proportion was significantly higher than that in older patients (≥ 65 years); it was most obvious in young patients (27%). A reason for this finding may be related to diabetes. Diabetes is an independent predictor of sudden cardiac death in patients on hemodialysis [16]. Among the deceased patients in our study, sudden death accounted for a higher proportion of patients with diabetes (20.0%) than patients without diabetes (16.3%). Moreover, the prevalence of diabetes was higher in young and middle-aged patients (53.2%) than in older patients (45.6%). Another reason may be that cerebrovascular disease also accounted for a high proportion of death in young and middle-aged patients in the present study, with the majority having cerebral hemorrhage (88.5%), which was also an important cause of sudden death. Therefore, it was possible that some unexpected deaths in young and middle-aged patients caused by cerebrovascular accidents were reported as “sudden death” or “sudden cardiac death” because of a lack of autopsy confirmation. In a previous autopsy study involving 35 Japanese patients on hemodialysis with sudden death [17], there were 9 cases of stroke, which accounted for 25.7% of patients, and was basically equivalent to the proportion of cardiovascular disease (10 cases, 28.6%). In addition, 125 sudden death patients in our study were caused by hyperkalemia, of which more than half (52.8%) were young and middle-aged patients. It was also observed in our clinical practice that this population was more likely to have interdialytic volume overload and hyperkalemia. Hyperkalemia causes cardiac depression; volume overload leads to a high ultrafiltration rate during dialysis, which is prone to hypotension, ischemia of other end-organs, and syncope; meanwhile, abrupt fluctuations in electrolytes (e.g., potassium, calcium) during dialysis. All these factors increase the risk of sudden cardiac death [18]. It should also be mentioned that the proportion of sudden deaths in 2020 was higher than the proportions in 2019 and previous years. The reasons maybe related to the COVID-19 epidemic: From the end of 2019 to the beginning of 2020, COVID-19 began to erupt in Wuhan, China. Because of the implementation of strict control measures and cooperation in Beijing, there was a very small number of COVID-19 infected individuals in 2020 (982 infectors and 9 deaths) However, Beijing experienced a SARS epidemic in 2003 that left a deep impression on the people of Beijing. Therefore, when the COVID-19 epidemic began, many people in Beijing rarely went out because of fear of contracting the virus, and many patients with CKD who were being followed up on a monthly basis did not come to the outpatient clinic for a long time (even if this resulted in medication discontinuation). Even patients on regular dialysis reduced their frequency of seeking medical treatment, which meant that many people tolerated symptoms without seeking medical help. This may have led to delays in the diagnosis and treatment of some life-threatening diseases, especially cardiovascular diseases, leading to an increased probability of sudden death among patients on dialysis.

Diabetes increases systemic vascular inflammation. Patients on MHD with diabetes have a higher risk for cardiovascular disease [19] and a higher relative risk for death [20] compared with patients on MHD without diabetes. Our study showed that patients on MHD with DN had the highest annual mortality rate, which was consistent with the results of other domestic studies [15]. In addition, the proportions of death from cardiovascular disease, sudden death, and infection were higher in patients with diabetes than in patients without diabetes. Since 2007, the number of patients on hemodialysis with diabetes in Beijing has increased annually, and diabetes has become the leading cause of incident hemodialysis [21]. This is also an important reason for the increase in the proportion of death from infection compared with that in the previous period. In our study, the proportion of deceased patients on MHD with diabetes who had a dialysis vintage ≥ 10 years was only 6.2%, which was significantly lower than that of patients without diabetes (18.0%). These findings all suggest high mortality and short survival in patients on MHD with diabetes.

China is currently experiencing an aging society, and more than half of the patients with ESRD starting dialysis are middle-aged and older [1]. In recent years in Beijing, the proportion of new patients on hemodialysis aged ≥ 60 years has increased annually [21]. In our study, the median age of death was 70.0 (60.7–78.9) years. With increasing age, the functions of various organs decline and the ability to resist disease reduces; thus,the incidence of death from infection in older patients is often high. Our study showed that 24.5% of deceased patients aged over 75 years died of infection, which was the leading cause of death in this age group, and pneumonia accounted for 73.6%. However, even with the effect of aging, the dialysis vintage of deceased patients still slowly increased annually (from 3.4 to 4.0 years). The proportion of deceased patients with a dialysis vintage ≥ 5 years increased compared with that reported for 2007–2010 (38.2%vs.30.2%) [13]. This may be related to the annual improvements in the quality of hemodialysis in Beijing.

The present study had some limitations. First, there were missing values. This study is representative if the deletions were random but may be biased if the deletions were nonrandom. Second, this was a retrospective study, the cause of death was self-reported by each hemodialysis center, and there may be errors in the judgment of the cause of death.

## Conclusion

The results of our study have good guiding significance for clinical practice. Comprehensive management should be performed for patients on MHD with diabetes, including life guidance, specialist care, and prevention and control of cardiovascular disease and infections. In older patients, prevention and control of infection, especially respiratory infection, should receive more attention to further reduce mortality. Sudden death accounted for a high proportion of deaths in young and middle-aged patients, suggesting that management of this population needs to be more detailed and individualized, such as good control of blood pressure and blood sugar, and correcting abnormalities of calcium, phosphorus, and parathyroid hormone to reduce the occurrence and progression of vascular calcification. It is also necessary to strengthen the publicity and education regarding weight and diet control for patients during interdialysis. Multiple factors should be considered when formulating and adapting patients’ dialysis regimens, including their comorbidities, symptoms, underlying residual kidney function, and lifestyle patterns, to reduce the risk of sudden death in this population.

## Data Availability

The datasets generated and analysed during the current study are not publicly available due to the access to data is restricted in accordance with government mandates, but are available from the corresponding author on reasonable request.

## Abbreviations

MHD: maintenance hemodialysis
ESRD: end-stage renal disease
CGN: chronic glomerulonephritis
DN: diabetic nephropathy
HN: hypertensive nephropathy
IN: interstitial nephropathy
PKD: polycystic kidney disease
PD: peritoneal dialysis
KT: kidney transplantation
CKD: chronic kidney disease
BJBPQCIC: Beijing Blood Purification Quality Control and Improvement Center
PDC: patient–doctor contact
